# Atypical Infections in Tsunami Survivors

**DOI:** 10.3201/eid1110.050715

**Published:** 2005-10

**Authors:** Christian Garzoni, Stéphane Emonet, Laurence Legout, Rilliet Benedict, Pierre Hoffmeyer, Louis Bernard, Jorge Garbino

**Affiliations:** * University Hospitals of Geneva, Geneva, Switzerland

**Keywords:** tsunami, Southeast Asia, infection, dispatch

## Abstract

After a tsunami hit Asia in December 2004, 2 survivors had severe infections due to multidrug-resistant and atypical bacteria and rare fungi weeks afterwards. Treating these infections is challenging from a clinical and microbiologic point of view.

After a tsunami hit a large part of Southeast Asia in December 2004, >200,000 people died and several hundred thousand were severely injured. First aid was provided in local hospitals under difficult conditions. Most patients had multiple fractures, soft-tissue injuries, and complications from near-drowning events (*1**,**2*). Acute complications did not pose diagnostic problems; after emergency situations were resolved, patients were seen in other healthcare facilities, and foreign tourists were repatriated. Two Swiss tourists were treated in Thailand and then transferred to our hospital. Severe infections that were caused by multidrug-resistant bacteria and, subsequently, unusual fungal and mycobacterial infections developed in both of the patients.

## The Study

### Case 1

A previously healthy, 59-year-old man was treated in Thailand for aspiration pneumonia complicated by multi-organ failure and septic shock, necessitating mechanical ventilation and hemodialysis. *Acinetobacter baumannii* (resistant to all penicillins, cephalosporins, aminoglycosides, and trimethoprim-sulfamethoxazole) and *Escherichia coli* were found in cultures from bronchoalveolar lavage performed 48 h after a near-drowning episode and were caused by massive bronchoaspiration. Both bacteria strains were sensitive to imipenem-cilastatin and ciprofloxacin. Because of persistent fever and cough after 2 weeks of treatment with ciprofloxacin and imipenem-cilastatin, a computed tomographic scan of the chest was performed; an abscess was seen in the left lung. Culture of the abscess yielded an *Acinetobacter* sp. that was resistant to imipenem-cilastatin; drug was changed to ampicillin-sulbactam.

Three weeks later, the patient was transferred to our hospital because he had several pulmonary abscesses and an empyema that required repetitive drainage. A lobectomy of the left necrotic lobe was performed. After 6 weeks of treatment with piperacillin-tazobactam, the patient was discharged from the hospital. One month later, the patient returned to the hospital with back pain with no previous spinal pathology. Paravertebral collection showed spondylodiscitis (T8–T9), caused by *Scedosporium apiospermum*. After surgical drainage, the spine was immobilized with an external corset, and treatment with voriconazole (200 mg IV twice daily) was started. The patient progressed well clinically and had no neurologic complications.

### Case 2

A previously healthy, 51-year-old woman with deep cutaneous wounds of the legs, multiple pelvic fractures, and a ruptured bladder lay immobilized in mud for >24 h. At the local hospital, after her hemodynamic status was stabilized, a laparotomy confirmed the bladder injury. Surgical debridement of the patient's wounds was performed. The patient was repatriated and admitted to our hospital on December 31. The soft-tissue wounds were infected with multidrug-resistant bacteria: *A. baumannii* (resistant to all penicillins, cephalosporins, aminoglycosides, fluoroquinolones, and trimethoprim-sulfamethoxazole; sensitive only to colistin); *Stenotrophomonas maltophilia* (sensitive only to piperacillin-tazobactam); and *Achromobacter xylosoxidans* (sensitive to piperacillin-tazobactam, imipenem-cilastatin, ciprofloxacin). Penicillin-resistant *Enterococcus faecium* and *Pseudomonas aeruginosa* were also seen in the cultures. The wounds that were colonized by multidrug-resistant bacteria were treated with aggressive surgical debridement and local instillation of colistin. A computed tomographic scan of the chest was performed because dyspnea developed. The scan showed bilateral infiltration, and pneumonia was confirmed. To minimize further selection pressure by antimicrobial drugs and treat the concomitant pneumonia caused by *Pseudomonas* sp., targeted therapy with piperacillin-tazobactam was administered for 14 days. Pelvic open fractures were in direct contact with urine. The bladder injury was repaired surgically, and urine specimens showed *A. baumannii* (sensitive only to colistin, which was used for bladder irrigation) and *E. faecium* when cultured. Because the pelvic open fractures were in contact with infected urine and stable, orthopedic surgeons decided that bed rest was the treatment of choice. The pelvic fractures healed without sequelae. However, an abscess developed on the patient's thigh 2 weeks after admission. *Nocardia africanum* was cultured from the samples taken at the time of intervention. The patient was treated with trimethoprim-sulfamethoxazole for 10 weeks.

On week 8 of hospitalization, persistent fever and progressive alteration of consciousness developed in the patient. A computed tomographic scan showed an intracerebral abscess with hydrocephalus (Figure). The abscess was drained surgically, and a ventricular-peritoneal drain to treat aresorptive hydrocephalus was subsequently put in place. The abscess cultures showed *S. apiospermum*, and voriconazole (4 mg/kg IV twice a day) was started. The clinical course was slow but favorable. Magnetic resonance imaging performed after 3 months of voriconazole treatment showed a reduction in the dimensions of the abscess. Voriconazole treatment was scheduled for 6 months or until resolution of the cerebral lesion.

**Figure:**
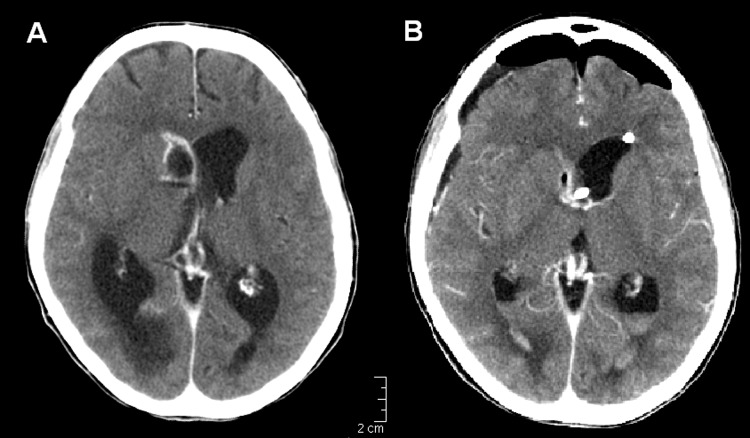
Brain abscess caused by Scedosporium apiospermum (patient 2). A) Images from contrast-enhanced computed tomographic scan show a ring-enhancing lesion in the head of the nucleus caudatus (2 × 1.5 cm) bulging in the right lateral ventricle with concomitant aresorptive hydrocephalus. B) Control computed tomographic scan after surgical drainage and placement of ventricle-peritoneal drainage.

Three months after the tsunami, the patient still had a residual open wound on the tibial area of the leg. Because of healing difficulties, even with antimicrobial drug treatment, specialized tests were conducted on the wound for a resistant or atypical pathogen. Special cultures for mycobacteria permitted the growth of *Mycobacterium chelonae*, which was sensitive to amikacin and clarithromycin and resistant to imipenem-cilastatin, fluoroquinolones, and trimethoprim-sulfamethoxazole. Magnetic resonance imaging results excluded osteomyelitis. Treatment with clarithromycin was initiated and surgical debridement was accomplished.

## Conclusions

These 2 patients had unusual and severe lesions, pathogens that were difficult to treat, and complications that could be encountered in tsunami survivors. The situation constitutes an exceptional event, and several factors put the survivors at risk in the short- and the long-term. In the first days after the event, survivors were likely to have bacterial complications of soft tissue and bone injuries and aspiration pneumonia. Some case reports that were recently published described less frequent infections, such as cutaneous mucormycosis (*3*) or unusual pathogens, such as *Bacillus pseudomallei* (*2*), outside the affected region.

To observe multiple infections in the same patient caused by several different multidrug-resistant bacteria is unusual in clinical practice. Treatment is complex, and additionally, using broad spectrum antimicrobial drug therapy in patients with high bacteria count may lead to resistance. In these 2 patients, incorporating optimal antimicrobial drug therapy to treat all isolated germs was very difficult. Aggressive surgical intervention was essential to ensure the efficacy of treatment.

Most of the tsunami survivors who experienced near-drowning events remained in unclean and traumatic conditions without receiving any immediate medical care (*4*) for several hours. Near-drowning is a rare event; therefore, experience is limited in dealing with the resulting complications (*5*). Tepid, salty, and brackish water was inhaled and ingested. Patients lay for several hours or days in warm, stagnant water and slush; normally poorly virulent environmental bacteria, fungi, and amoebae found the ideal conditions to colonize in open wounds and bone fractures and disseminate to other body sites.

*S. apiospermum* is a ubiquitous saprophytic fungus that rarely causes invasive infections in an immunocompetent host. In addition to anatomic barrier alterations, such as burns, trauma, or neurosurgery, near-drowning events promote favorable conditions for elevated numbers of fungal infections. The potent activity of voriconazole against this fungus and its availability, with good penetration of the hematoencephalic barrier, may increase the chance of recovery and survival. Otherwise, the prognosis is poor (*6*). Both patients reported here have shown good clinical response to treatment.

The second patient had a cutaneous infection with *M. chelonae*, a rapidly growing mycobacteria that is ubiquitous in soil and water worldwide. Generalized infections are seen mainly in immunosuppressed patients; however, isolated cutaneous infections have been reported in immunocompetent patients. The diagnosis may be difficult because of the necessity of obtaining specific mycobacterial cultures. In addition, the treatment may be complex, as *M. chelonae* is among the most resistant mycobacteria, and adaptation of therapy according to sensitivity tests is mandatory (*7*).

A number of conditions found in tsunami survivors could render infection treatment extremely difficult. These conditions include the large number of relatively rare environmental pathogens that result from particularly traumatic exposure; extensive soft tissue and internal injuries; the possible presence of multidrug-resistant bacteria, atypical bacteria, and fungal infections; and initial treatment administered with limited resources and under difficult emergency conditions. All these factors contribute to the severity of complications and difficulties in treating infections in tsunami survivors. In addition, an optimal infection control policy is required in managing these patients to prevent the spread of imported nosocomial infections (*8*).

This article aims to raise awareness about the possibility of unusual complications and highly-resistant microorganisms that can lead to extensive illness and death in tsunami survivors. Most clinicians are probably unfamiliar with the pathogens that would be found under these conditions. Therefore, every tsunami survivor should be considered a high-risk patient, even months after the event. The severe and atypical infections they may have pose challenges for diagnosis and treatment, even for experienced infectious disease specialists.
